# Efficacy and Safety of Mucopolysaccharide Polysulfate Cream for Non-Exudative Eczema: A Systematic Review and Meta-Analysis

**DOI:** 10.3389/fmed.2021.788324

**Published:** 2021-12-24

**Authors:** Ming Li, Yan Li, Lujing Xiang, Linfeng Li

**Affiliations:** Department of Dermatology, Beijing Friendship Hospital, Capital Medical University, Beijing, China

**Keywords:** eczema, mucopolysaccharide polysulfate, hirudoid, meta-analysis, efficacy, safety

## Abstract

**Background:** Mucopolysaccharide polysulfate (MPS) cream as a moisturizer is widely applied to treat eczema, and a lot of clinical trials have demonstrated its efficacy and safety. However, there is no further research to collect and analyze these studies.

**Objective:** This meta-analysis aimed to assess the efficacy and safety of MPS cream as monotherapy or add-on therapy for non-exudative eczema.

**Methods:** Ten databases were searched to identify the eligible randomized controlled trials (RCTs) from their inception to July 31, 2021. Revman 5.3 software was used for the meta-analysis.

**Results:** A total of eligible 20 studies were included. Among the 20 studies, 2 studies compared MPS cream with other moisturizers, 14 compared MPS cream plus topical corticosteroids (TCS) with TCS alone, and 4 compared with MPS cream plus tacrolimus ointment with tacrolimus ointment alone. The pooled results demonstrated that MPS cream had a higher total efficacy rate [Risk ratio (RR) 1.21, 95% CI: 1.12 to 1.30, *P* < 0.00001], a lower recurrence rate (RR 0.44, 95% CI: 0.26 to 0.74, *P* = 0.002) and a lower pruritus score [mean difference (MD) −1.78, 95% CI: −2.16 to −1.40, *P* < 0.00001] than urea cream or vaseline ointment. Moreover, in comparison with TCS or tacrolimus ointment alone, the combination treatment performed better in terms of total efficacy rate, total symptom score, recurrence rate, and pruritus score. For safety, the skin adverse events were mild, and MPS cream as monotherapy or add-on therapy did not increase the risk of skin adverse events.

**Conclusions:** MPS cream as monotherapy or add-on therapy could provide a good effect for treating non-exudative eczema with mild and tolerable skin adverse events. However, due to the suboptimal quality of the included studies, high-quality and large-sample RCTs are needed in the future for update or validation.

**Systematic Review Registration:** PROSPERO (https://www.crd.york.ac.uk/PROSPERO/), identifier: CRD42021265735.

## Introduction

Eczema, also known as atopic eczema or atopic dermatitis, is a common chronic skin disease characterized by itch and a wide spectrum of clinical signs, such as erythema, papules, vesicles, crust, lichenification, and dry skin (1). About 15–20% of children and 1–3% of adults are affected around the world (2). The pathophysiology of eczema is complex and results from complex interactions between genetic and environmental factors. Although not life-threatening, eczema has a negative impact on a patient's life quality, which results in serious public health problems and economic costs (3). In many guidelines for the treatment of eczema, both topical corticosteroids (TCS) and topical calcineurin inhibitor (TCI), as anti-inflammatory treatments, are recommended to reduce skin inflammation, and oral antihistamines could be applied to relieve pruritus if necessary. On the other hand, due to skin barrier dysfunction and dry skin in eczema patients, emollient therapy is important and basic. It could improve skin barrier function and reduce skin susceptibility to irritants. Different moisturizer products have different mechanisms of storing skin barriers. For example, urea and glycerol can promote stratum corneum hydration, and vaseline can reduce evaporation (4–6).

Mucopolysaccharide polysulfate (MPS) cream, also termed hirudoid cream, is a heparinoid-containing product. Besides superficial phlebitis and skin contusion, MPS cream has been widely used as an effective moisturizer to manage a variety of skin conditions, including eczema, psoriasis, post-operative ecchymosis and edema, radiation dermatitis, and senile xerosis (7–11). A questionnaire survey from Japan found that most enrolled atopic eczema patients (95.1%, 98/103) used heparinoid mucopolysaccharide creams or lotions for moisturizing the skin, and the application for one month significantly improved skin dryness, pruritus, and eczematous skin (12). Moreover, heparinoid preparation is recommended as skincare against dry skin in the Japanese guidelines for atopic eczema (6). Several clinical studies in China also have demonstrated that MPS cream is effective and safe for the treatment of eczema in both children and adults (13, 14).

To the best of our knowledge, there has been no systematic review to summarize the efficacy and safety of MPS cream on non-exudative eczema. Therefore, the current study aimed to perform a systematic review and meta-analysis of all published randomized controlled trials (RCTs) to evaluate the efficacy and safety of MPS cream as monotherapy or add-on therapy for non-exudative eczema and to provide recommendations for clinical practice.

## Materials and Methods

This study was conducted in accordance with the Preferred Reporting Items for Systematic reviews and Meta-Analyses (PRISMA) guidelines and registered with PROSPERO (CRD 42021265735) (15).

### Databases and Search Strategy

Two researchers (ML and YL) searched the following ten databases from their inception to July 31, 2021, including PubMed, the Cochrane Library, Embase, Web of Science, ClinicalTrials.gov (https://www.clinicaltrials.gov/), China National Knowledge Infrastructure (CNKI), WangFang Database (WangFang), Chinese Biomedical Literature (CBM), Chongqing VIP (CQVIP), and Chinese Clinical Trial Registry (ChiCTR, https://www.chictr.org.cn). The search strategy was as follows: {[eczema (MeSH Terms) OR dermatitis (MeSH Terms) OR lichen simplex chronicus (MeSH Terms)] OR [eczema (Title/Abstract) OR dermatitis (Title/Abstract) OR lichen simplex chronicus (Title/Abstract)]} AND [mucopolysaccharide polysulfate (Title/Abstract) OR hirudoid (Title/Abstract) OR heparinoid (Title/Abstract) OR Xi Liao Tuo (Title/Abstract) OR Xiliaotuo (Title/Abstract)].

### Study Selection

The participants, interventions, comparisons, outcomes, and study design (PICOS) criterion was used to establish the inclusion criteria.

#### Types of Participants

The patients were diagnosed with non-exudative eczema, regardless of age and gender.

#### Types of Interventions

The participants in the experimental groups were treated with MPS cream alone or MPS cream combined with TCS or TCI, such as tacrolimus ointment (TAC-O) and pimecrolimus cream. If necessary, oral antihistamines could be applied to relieve itch.

#### Types of Comparisons

The participants in the control groups were treated with placebo, other moisturizers (e.g., urea, glycerol, and vaseline), TCS, or TCI alone. Oral antihistamines could be used when they were needed to alleviate pruritus.

#### Types of Outcomes

The primary outcomes were total efficacy rate (TER), total symptom score (TSS), and recurrence rate. TSS was scored based on the severities of lesion morphology, lesion area, and pruritus symptom. TER was the proportion of participants with the improvement of TSS ≥ 60% or 70% of baseline at the end of the treatments.

The second outcomes included pruritus score, tests for skin barrier function, the levels of cytokines in serum, and adverse events (AEs). The tests for skin barrier function consisted of trans-epidermal water loss (TEWL), stratum corneum hydration, and epidermal sebum content. The cytokines in serum included IL-2, IL-4, IL-10, and IFN-γ. For skin AEs, itch, pain, tingling, burning, etc. belong to skin inflammatory reactions, and skin non-inflammatory reactions included skin infection, atrophy, hyperpigmentation, and telangiectasia.

#### Types of Study Design

RCTs published in Chinese or English were included.

Animal experiments, case reports, conference presentations, reviews, expert opinions, duplicates, and RCTs that compared MPS cream with TCS or TCI alone were excluded

The titles and abstracts of the retrieved studies were assessed by two independent authors (ML and YL), and the full texts of potentially eligible studies were screened to identify the included studies. Any disagreements were settled by consulting the third author (LJX).

### Data Extraction and Quality Assessment

Two authors (ML and YL) independently extracted the data from the eligible studies, including the first author, publication year, sample size, gender, age-range, interventions, comparisons, and outcomes. The methodological quality of the eligible studies was evaluated by two authors (ML and YL) independently based on the Cochrane Risk of Bias tool (16). The seven items were as follows: random sequence generation, allocation concealment, blinding of participants and personnel, blinding of outcome assessment, incomplete outcome data, selective reporting, and other bias. In this study, the baselines of eczema severity between two groups were considered as the source of other bias. The judgment of each item included low, unclear, or high risk of bias. Disagreements were resolved through discussion with the third author (LJX).

### Data Synthesis and Analysis

All statistical analyses were conducted by using the Review Manager 5.3 software (Copenhagen: The Nordic Cochrane Centre, The Cochrane Collaboration, 2014). Dichotomous data were expressed as risk ratio (RR) with 95% confidence interval (CI), whereas continuous data were expressed as mean difference (MD) or standardized mean difference (SMD) with 95% CI. The heterogeneity across studies was evaluated by using the *I*^2^ statistic. When *I*^2^ was ≥ 50%, indicating significant heterogeneity, the random-effect model was used. When *I*^2^ was <50%, indicating no heterogeneity, the fixed-effect model was performed. If 10 studies or more were involved in the same outcome, the funnel plot was used to assess the publication bias. Two-sided *P* < 0.05 was considered statistically significant.

## Results

**Figure 1:**
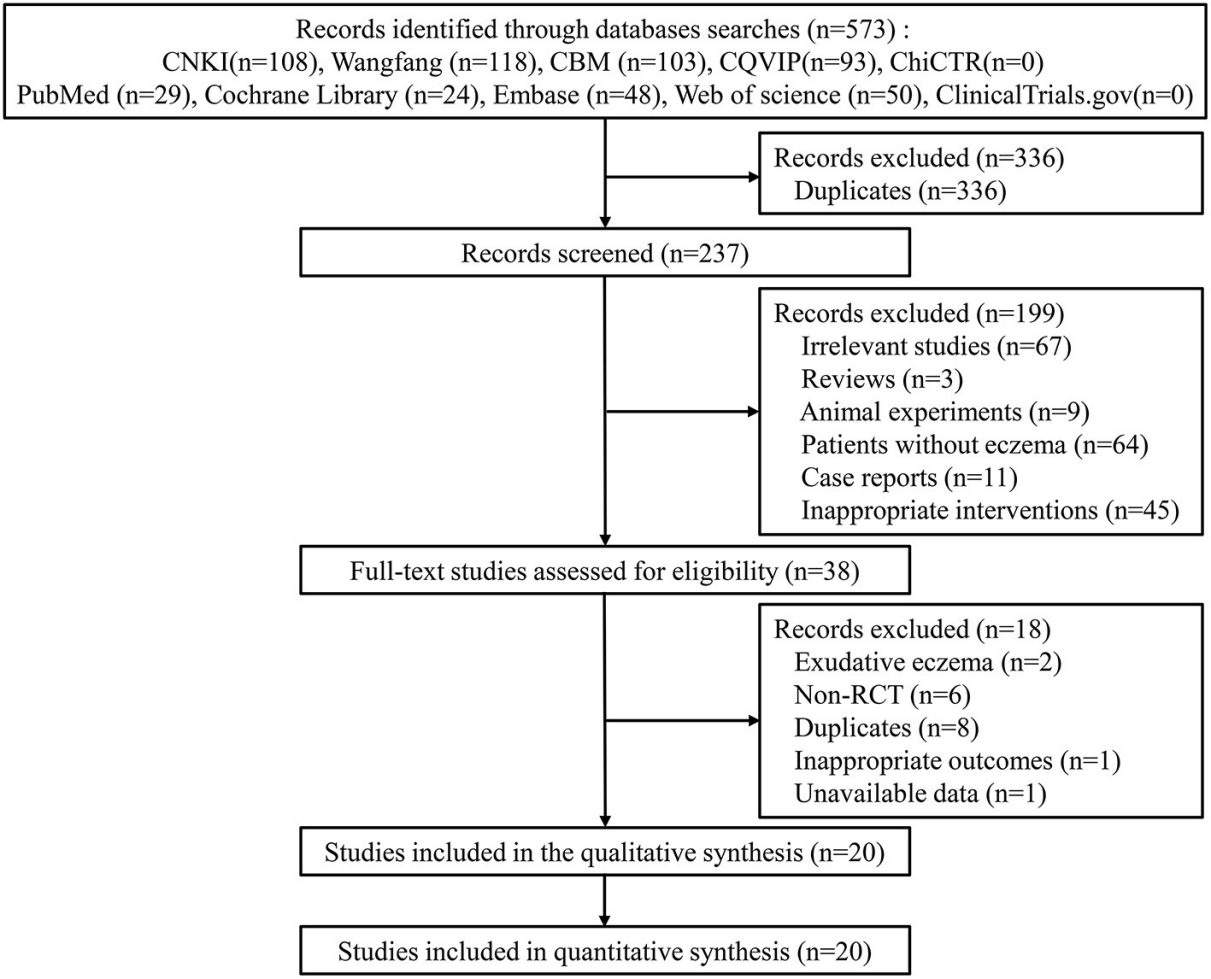
The flow diagram of the included studies in the meta-analysis.

The search process yielded 573 studies from ten databases. After eliminating duplicates, 237 studies were screened for eligibility, and 199 studies were excluded due to the titles and abstracts. A total of 38 full-text studies were reviewed for eligibility, and 18 studies were excluded. Finally, 20 studies were included for the meta-analysis (17–36) (Figure 1).

### Study Characteristics

All studies were conducted in China. Only one study was published in English (23), while the rest were published in Chinese. Sample sizes ranged from 60 to 500 participants, and a total of 2,389 patients were involved. Two studies compared MPS cream with urea cream or vaseline ointment (17, 18), 14 studies compared MPS cream plus TCS with TCS alone (19–32), and four studies compared MPS cream plus TAC-O with TAC-O alone (33–36). The basic characteristics of the included studies were shown in Table 1.

**Table 1:** The basic characteristics of the included studies.

**Study ID**	**Sample size** **(male/female)**		**The range of age** **(years)**		**Duration of** **disease** **(months)**		**Interventions**		**Course of treatment (weeks)**	**Outcomes**
	**T**	**C**	**T**	**C**	**T**	**C**	**T**	**C**		
Tan et al. (17)	250 (120/130)	250 (118/132)	18–70	18–70	3–96	3–96	MPS cream b.i.d + Desloratadine citrate disodium tablet 8.8 mg/d	Urea cream b.i.d + Desloratadine citrate disodium tablet 8.8 mg/d	4	①③⑦
Yang et al. (18)	58 (29/29)	59 (31/28)	12–58	10–77	0.5–4	0.5–5	MPS cream b.i.d	Vaseline ointment b.i.d	2	①④⑦
Zhang et al. (19)	43 (26/17)	43 (25/18)	16–75	16–71	6–60	6–60	MPS cream t.i.d + Clobetasol propionate cream t.i.d + Levocetirizine oral liquid 5 mg/d	Clobetasol propionate cream t.i.d + Levocetirizine oral liquid 5 mg/d	2	①②④⑦
Li (20)	32 (20/12)	32 (19/13)	25–76	24–75	7.2–84	6–84	MPS cream b.i.d + Desonide cream b.i.d	Desonide cream b.i.d	4	④
Hu et al. (21)	41 (19/22)	41 (20/21)	NA	NA	NA	NA	MPS cream bid + Triamcinolone acetonide and econazole cream b.i.d + Loratadine tablet 10 mg/d	Triamcinolone acetonide and econazole cream b.i.d + Loratadine tablet 10 mg/d	4	①②④⑤
Shi (22)	46 (21/25)	41 (19/22)	19–57	18–53	2.5–14	2–13	MPS cream b.i.d + Desonide cream b.i.d	Desonide cream b.i.d	3	①⑦
Dang et al. (23)	90 (50/40)	90 (48/42)	0.167–2	0.167–2	NA	NA	MPS cream b.i.d + Desonide cream b.i.d	Desonide cream b.i.d	2	①②
Wang and Guo (24)	100 (62/38)	100 (57/43)	0.083–1	0.083–1	0.167–6	0.167–6	MPS cream b.i.d + Desonide cream b.i.d	Desonide cream b.i.d	2	①②⑦
Wang et al. (25)	48 (23/25)	47 (26/21)	0.083–1.583	0.083–1.417	0.25–6	0.25–6	MPS cream b.i.d + Hydrocortisone cream b.i.d	Hydrocortisone cream b.i.d	8	①②⑥
Liu (26)	49 (25/24)	49 (26/23)	25–78	26–79	12–48	24–60	MPS cream b.i.d + Desonide cream q.d	Desonide cream q.d	4	①⑦
Guo (27)	40 (21/19)	40 (18/22)	18–69	20–70	15.5–36.2	18.6–36.7	MPS cream b.i.d + Mometasone furoate cream b.i.d	Mometasone furoate cream b.i.d	4	①⑦
Xu et al. (28)	60	60	0.25–2	0.25–2	1–6	1–6	MPS cream b.i.d + Hydrocortisone butyrate cream b.i.d	Hydrocortisone butyrate cream b.i.d	2	①
Liu and Liu (29)	52 (27/25)	31 (16/15)	15–71	16–72	1.5–5	1.6–6	MPS cream b.i.d + Mometasone furoate cream q.d	Mometasone furoate cream q.d	2	①
Feng (30)	60	60	0.167–2	0.167–2	1–6	1–6	MPS cream b.i.d + Hydrocortisone butyrate cream b.i.d	Hydrocortisone butyrate cream b.i.d	2	①③⑦
Zhang et al. (31)	35 (22/13)	32 (19/13)	23–75	23–75	NA	NA	MPS cream q.d + Fluticasone propionate cream b.i.d	Fluticasone propionate cream b.i.d	4	①②④⑦
Xiao et al. (32)	36 (19/17)	32 (17/15)	45–75	45–75	6–240	6–204	MPS cream q.d + Halometasone cream q.d	Halometasone cream q.d	4	①⑦
Dong (33)	48 (21/27)	48 (23/25)	21–55	20–54	4–108	3–108	MPS cream q.d or b.i.d + Tacrolimus ointment b.i.d	Tacrolimus ointment b.i.d	4	①
Wang (34)	43 (18/25)	43 (17/26)	35–46	35–47	6–78	6–84	MPS cream b.i.d + Tacrolimus ointment b.i.d	Tacrolimus ointment b.i.d	4	①③⑥
Xiang (35)	50 (13/37)	50 (12/38)	21–55	21–56	5–60	4–60	MPS cream q.d + Tacrolimus ointment b.i.d	Tacrolimus ointment b.i.d	4	①⑦
Di and Xu (36)	30	30	18–55	18–55	1–36	1–36	MPS cream q.d + Tacrolimus ointment q.d	Tacrolimus ointment q.d	4	①⑦

*MPS, mucopolysaccharide polysulfate; NA, not available; T, test group; C, control group; q.d, once daily; b.i.d, twice daily; t.i.d, three times daily.*

*① Total efficacy rate; ② Total symptom score; ③ Recurrence rate; ④ Pruritus score; ⑤ Tests for skin barrier function; ⑥ The levels of cytokines in serum; ⑦ Adverse events*.

### Risk of Bias Assessment

**Figure 2:**
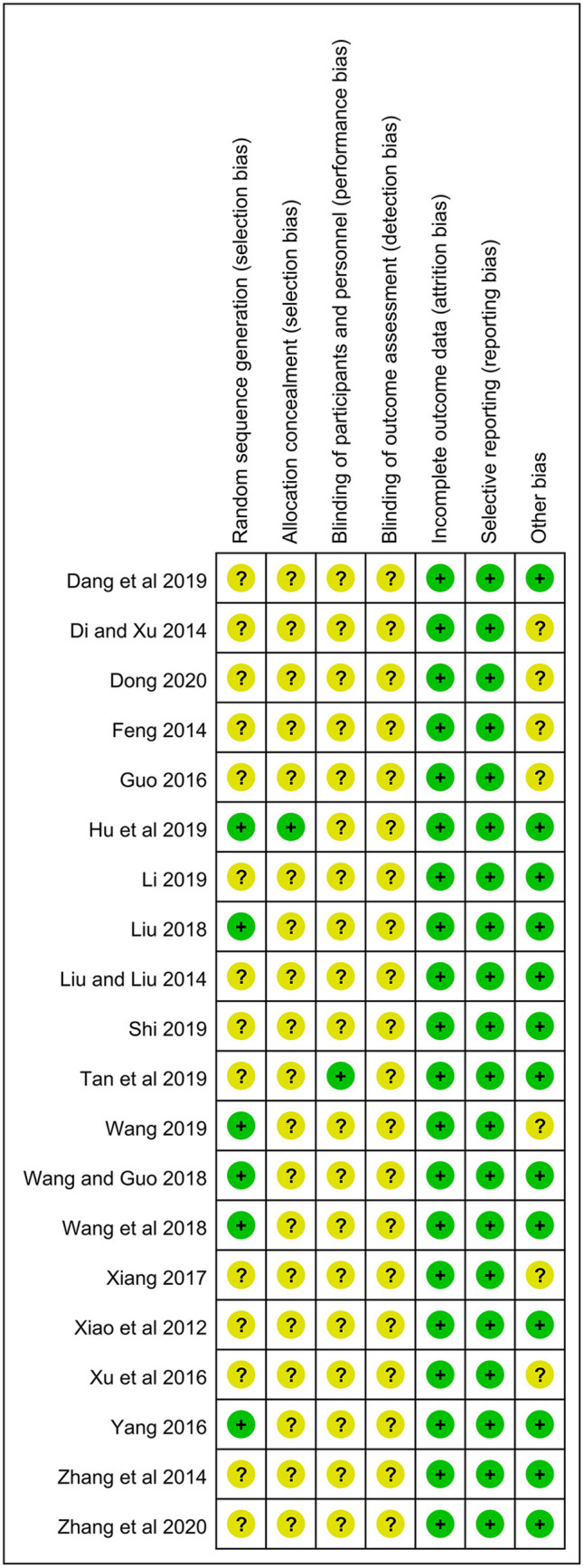
Risk of bias summary for the included studies.

All studies mentioned randomization. However, only six studies used the random number table and were rated as a low risk (18, 21, 24–26, 34). One reported the method of allocation concealment (21), and the rest lacked the corresponding information. Only one study was a double-blind RCT (17), and all studies did not describe the blinding of outcome assessment. All studies showed the complete outcome data, and the selective reporting was low risk in all studies. In terms of baseline of eczema severity, 13 studies had a low risk due to the comparability between two groups, while the remaining 7 studies were rated as an unclear risk because of the lack of relevant data (27, 28, 30, 33–36) (Figure 2).

### Primary Outcomes

#### Total Efficacy Rate

**Figure 3:**
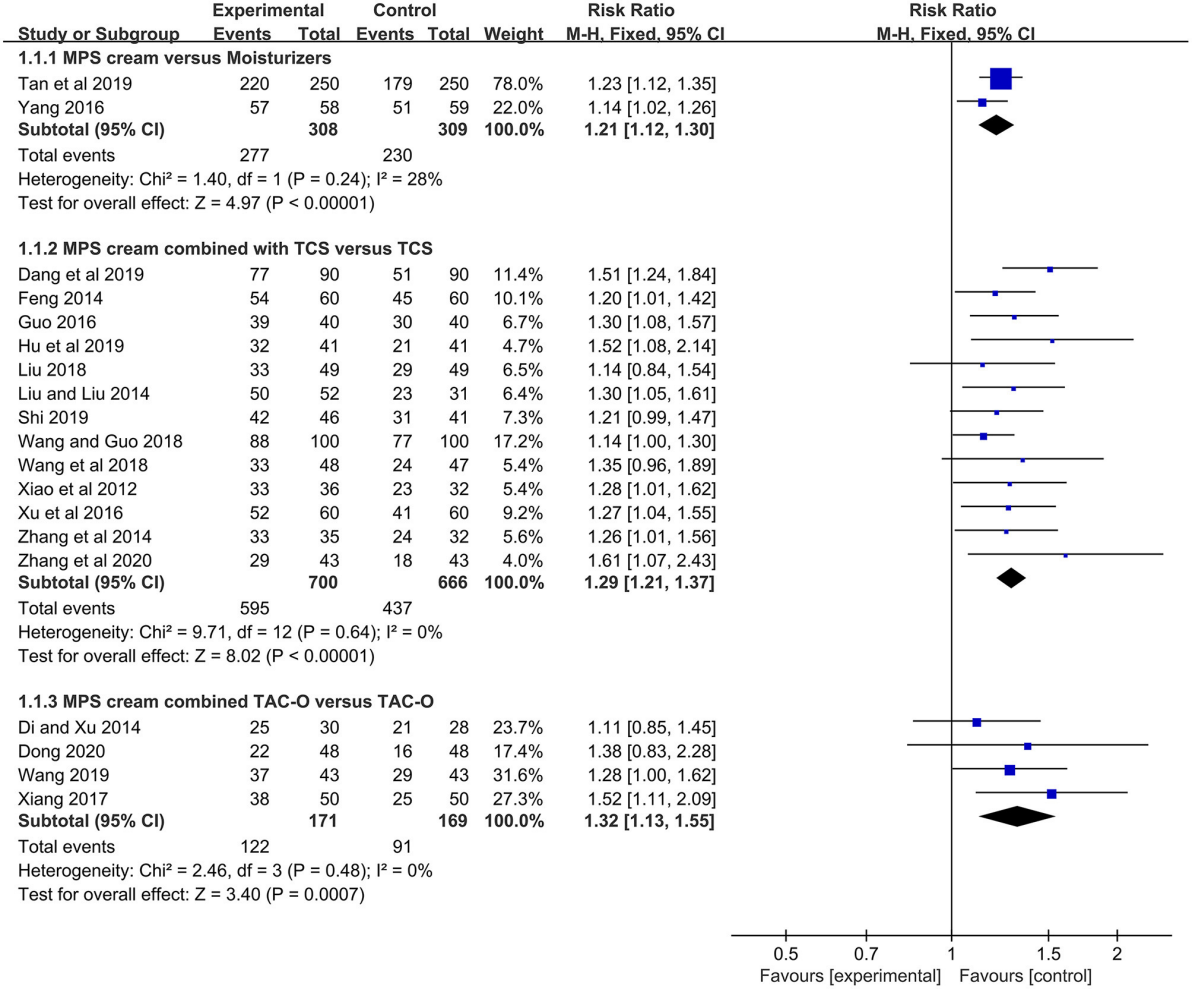
The forest plot for the total efficacy rate between MPS cream therapy and non-MPS cream therapy.

Two studies (*n* = 617) compared MPS cream with urea cream and vaseline ointment in terms of TER (17, 18). No heterogeneity was detected (*I*^2^ = 28%, *P* = 0.24), and a fixed-effect model was used. The pooled result showed that MPS cream had a significantly higher TER than other moiturizers (RR = 1.21, 95% CI: 1.12–1.30, *P* < 0.00001) (Figure 3).

In 13 included studies (*n* = 1,366) (19, 21–32), the TER between MPS cream combined with TCS and TCS alone was reported. Due to no heterogeneity (*I*^2^ = 0%, *P* = 0.64), a fixed-effect model was applied. The pooled result indicated that the combination therapy had a significantly higher TER compared with TCS monotherapy (RR = 1.29, 95% CI: 1.21–1.37, *P* < 0.00001) (Figure 3).

The TER between MPS cream combined with TAC-O and TAC-O alone was evaluated in 4 studies (*n* = 340) (33–36). A fixed-effect model was conducted because of no heterogeneity (*I*^2^ = 0%, *P* = 0.48). The pooled result revealed that the TER in the patients treated with MPS cream and TAC-O was prominently higher than that in the patients treated with TAC-O alone (RR = 1.32, 95% CI: 1.13–1.55, *P* = 0.0007) (Figure 3).

#### Total Symptom Score

**Figure 4:**
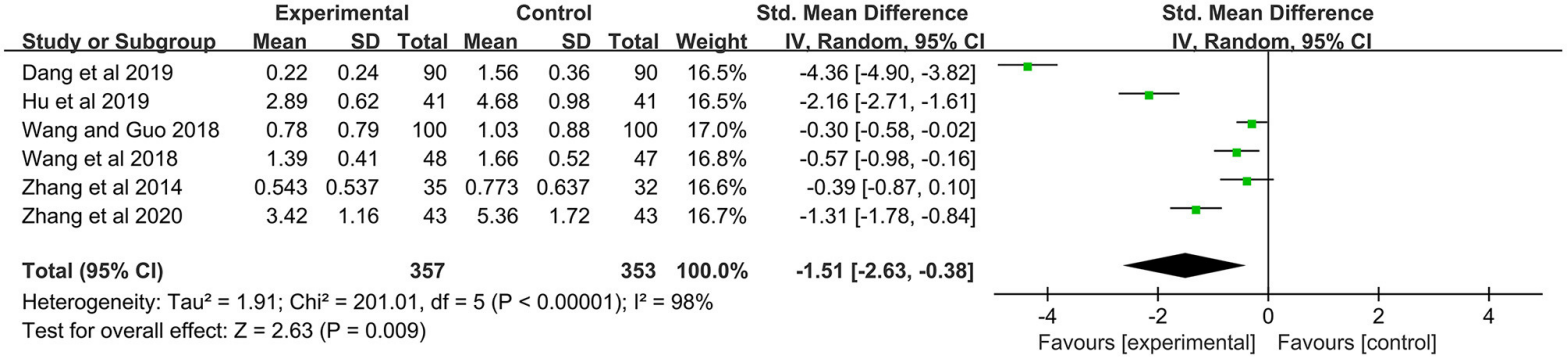
The forest plot for total symptom score between MPS cream combined with TCS therapy and TCS monotherapy.

The TSS was pooled from the data from 6 included studies (*n* = 710) which compared the combination of MPS cream and TCS with TCS alone (19, 21, 23–25, 31). Due to the different methods to assess eczema severity and a significant heterogeneity (*I*^2^ = 98%, *P* < 0.00001), SMD and the random-effect model were employed. The result showed that the combination therapy exerted a better treatment effect in terms of TSS, compared with TCS alone (SMD = −1.51, 95% CI: −2.63 to −0.38, *P* = 0.009) (Figure 4).

#### Recurrence Rate

**Figure 5:**
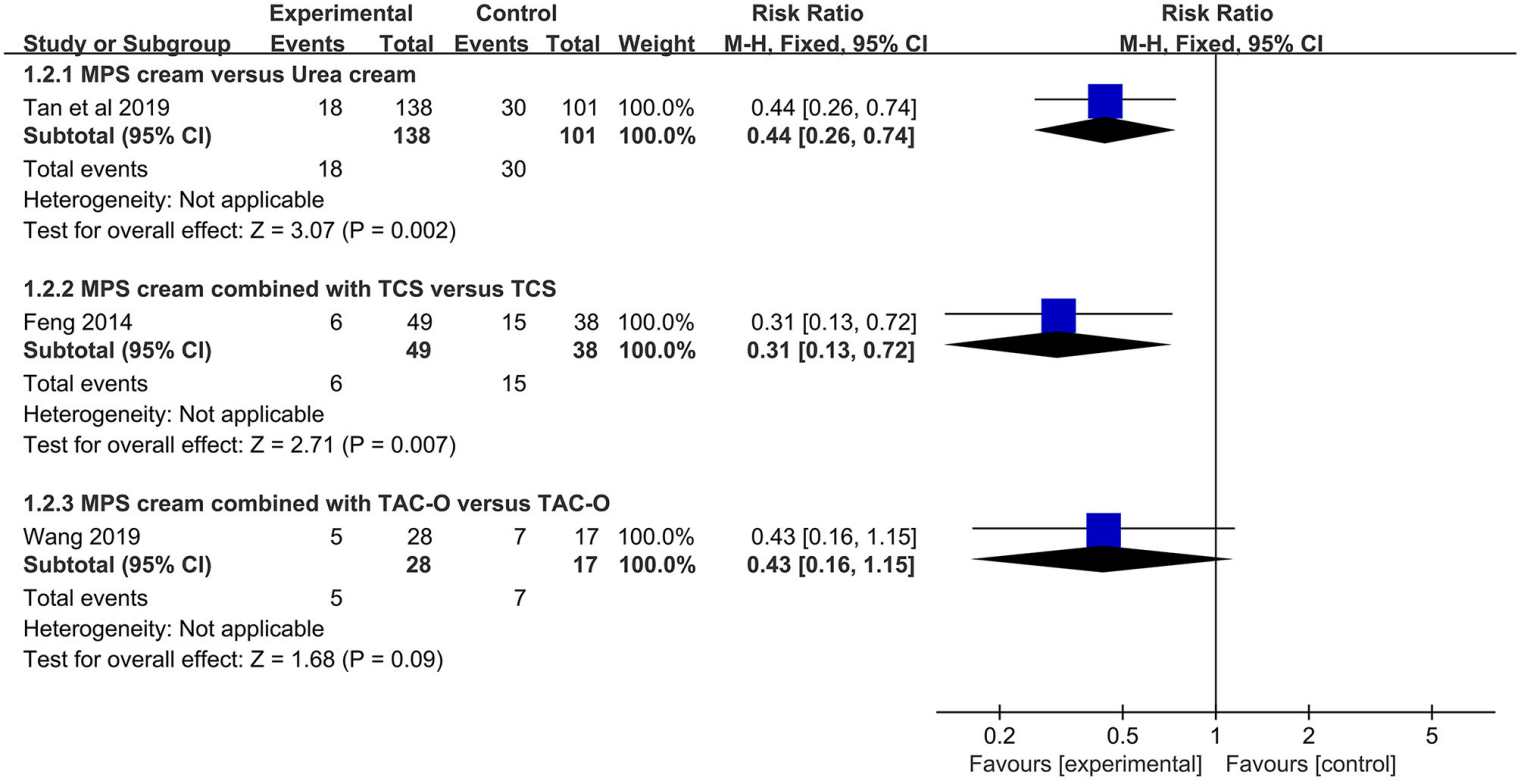
The forest plot for recurrence rate between MPS cream therapy and non-MPS cream therapy.

One study (*n* = 239) revealed that MPS cream could significantly decrease the recurrence rate compared with urea cream (RR = 0.44, 95% CI: 0.26–0.74, *P* = 0.002) (17), and another study (*n* = 87) showed that there was a significantly lower recurrence rate in the combination therapy group in comparison with the hydrocortisone butyrate cream alone group (RR = 0.31, 95% CI: 0.13–0.72, *P* = 0.007) (30). However, according to a small-sample study (*n* = 45) (34), there was no significant difference between MPS cream plus TAC-O and TAC-O alone with respect to recurrence rate (RR = 0.43, 95% CI: 0.16–1.15, *P* = 0.09) (Figure 5).

### Secondary Outcomes

#### Pruritus Score

One study (*n* = 117) compared MPS cream with vaseline ointment in terms of pruritus score (18). It was found that MPS cream had a significantly lower pruritus score than vaseline ointment (MD = −1.78, 95% CI: −2.16 to −1.40, *P* < 0.00001) (Supplementary Figure 1).

**Figure 6:**
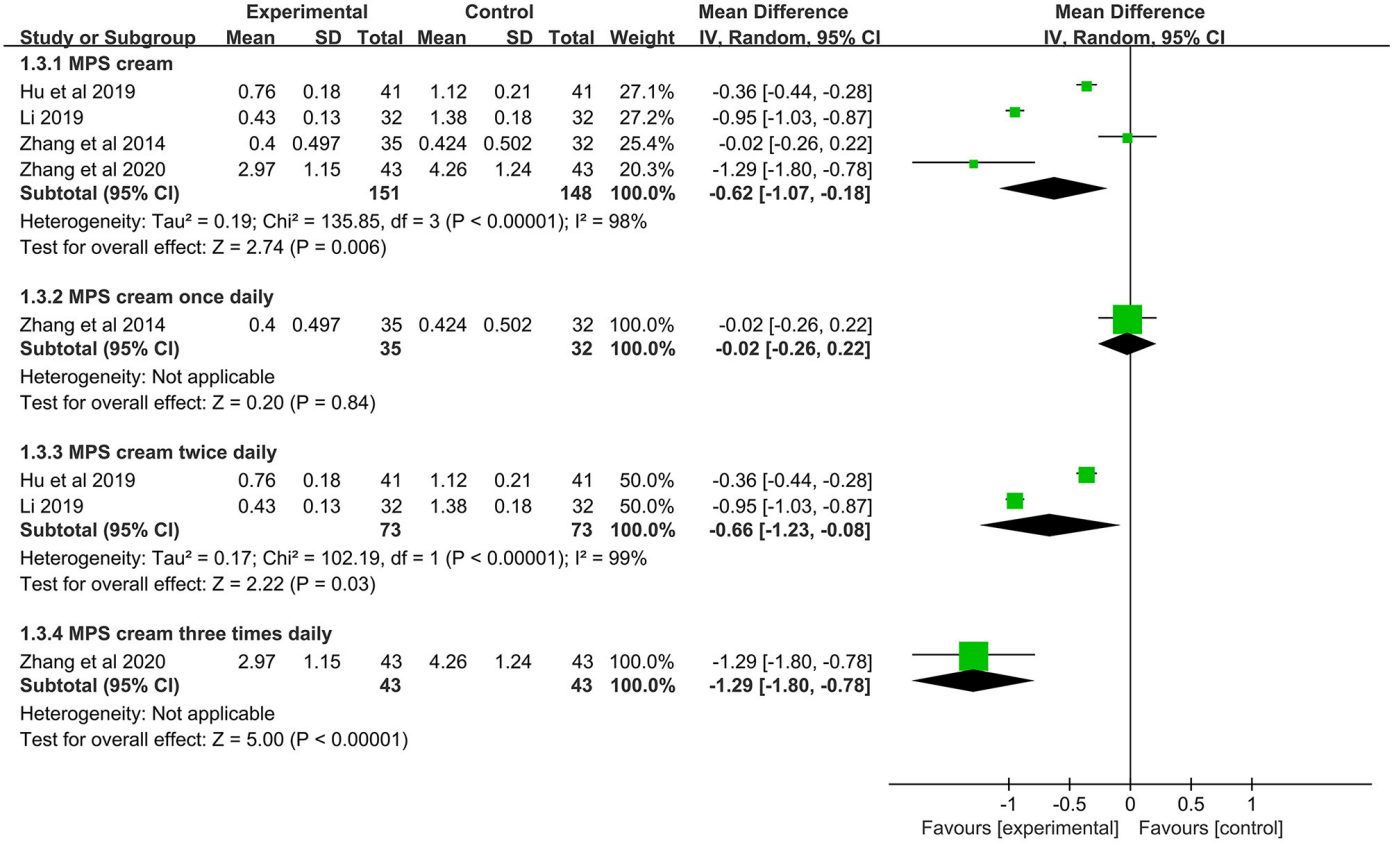
The forest plot for pruritus score between MPS cream combined with TCS and TCS monotherapy.

In 4 included studies (*n* = 299), the pruritus scores between MPS cream combined with TCS and TCS alone were measured (19–21, 31). Because of a significant heterogeneity (*I*^2^ = 98%, *P* < 0.00001), a subgroup analysis was conducted based on the frequency of application. The pooled result showed that MPS cream once daily combined with TCS did not significantly relieve pruritus compared with TCS monotherapy (MD = −0.02, 95% CI: −0.26 to 0.22, *P* = 0.84), but MPS cream twice daily plus TCS showed a superior pruritus score over TCS alone (MD = −0.66, 95% CI: −1.23 to −0.08, *P* = 0.03) and MPS cream three times daily plus TCS had the same result (MD = −1.29, 95% CI: −1.80 to −0.78, *P* < 0.00001) (Figure 6).

#### Tests for Skin Barrier Function

One study (*n* = 82) evaluated the function of MPS cream to restore skin barrier function (21). Compared with TCS monotherapy, MPS cream combined with TCS could significantly decrease TEWL (MD = −3.96 g·h^−1^·cm^−2^, 95% CI: −5.72 to −2.20 g·h^−1^·cm^−2^, *P* < 0.0001) and increase stratum corneum hydration (MD = 4.23%, 95% CI: 2.03–6.43%, *P* = 0.0002) (Supplementary Figure 2).

#### The Levels of Cytokines in Serum

One study (*n* = 95) measured the levels of IL-4, IL-10 and IFN-γ in the serum of infantile eczema (25). Compared with hydrocortisone cream monotherapy, MPS cream as an add-on treatment could significantly decrease the levels of IL-4 (MD = −14.76 pg/ml, 95% CI: −16.57 to −12.95 pg/ml, *P* < 0.00001) and IL-10 (MD = −2.82 pg/ml, 95% CI: −3.49 to −2.15 pg/ml, *P* < 0.00001), and increase the level of IFN-γ (MD = 8.44 pg/ml, 95% CI: 6.09–10.79 pg/ml, *P* < 0.00001) (Supplementary Figure 3).

The levels of IL-2, IL-4, and IFN-γ in the serum of adult eczema were measured in one study (*n* = 86) (34). In comparison of TAC-O monotherapy, the level of IL-4 was significantly decreased (MD = −4.83 pg/ml, 95% CI: −7.87 to −1.79 pg/ml, *P* = 0.002), while the levels of IL-2 (MD = 9.94 pg/ml, 95% CI: 6.19–13.69 pg/ml, *P* < 0.00001) and IFN-γ (MD = 6.21 pg/ml, 95% CI: 2.22–10.20 pg/ml, *P* = 0.002) were significantly increased in the MPS cream combined with TAC-O group (Supplementary Figure 4).

#### Adverse Events

Further results showed that 12 studies reported AEs of MPS cream during 2 to 4 weeks treatment (17–19, 22, 24, 26, 27, 30–32, 35, 36). A few cases with mild drowsiness and gastrointestinal discomfort were only reported in both groups treated with oral antihistamines, and they were considered to be uncorrelated to MPS cream. Some mild skin inflammatory reactions were observed in the MPS cream groups and the control groups, including erythema, mild burning, and tingling. These symptoms could disappear spontaneously after the treatment withdrawal. No skin non-inflammatory reaction was observed in both groups.

**Figure 7:**
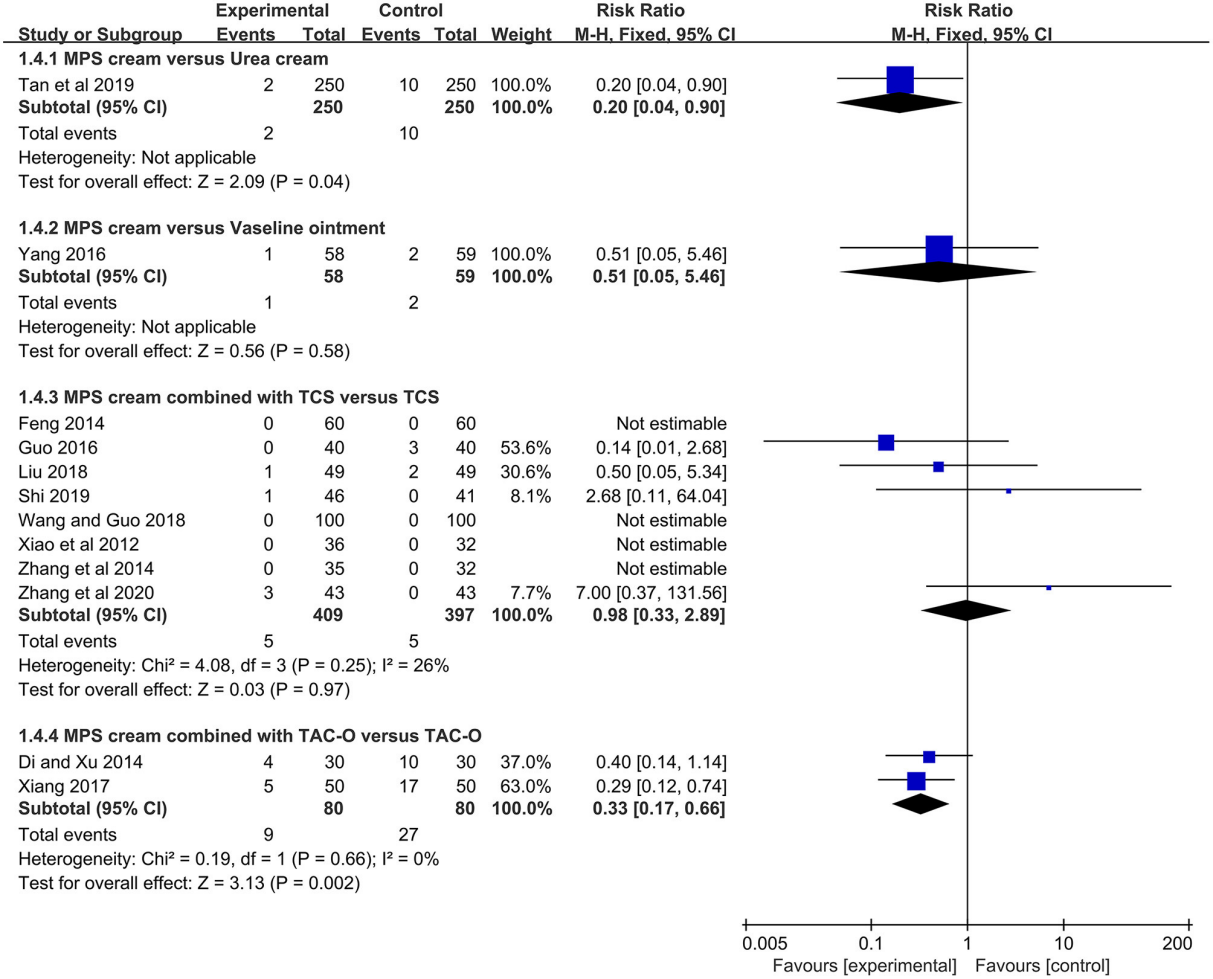
The forest plot for the incidence of skin inflammatory reactions between MPS cream therapy and non-MPS cream therapy.

One study (*n* = 500) showed that the MPS cream group had a significantly lower rate of skin inflammatory reactions than urea cream (RR =0.20, 95% CI: 0.04–0.90, *P* = 0.04) (17). In another study (*n* = 117) (18), the rates of skin inflammatory reactions between MPS cream and vaseline ointment were comparable (RR = 0.51, 95% CI: 0.05–5.46, *P* = 0.58) (Figure 7).

The skin inflammatory reactions in the MPS cream combined with TCS group were reported in 8 included studies (*n* = 806) (19, 22, 24, 26, 27, 30–32). Due to no heterogeneity (*I*^2^ = 26%, *P* = 0.25), a fixed-effect model was used. The pooled result showed that the combination therapy did not statistically increase the risk of skin inflammatory reactions compared with TCS monotherapy (RR = 0.98, 95% CI: 0.33–2.89, *P* = 0.97) (Figure 7).

Two studies (*n* = 160) reported skin inflammatory reactions between MPS cream combined with TAC-O and TAC-O alone (35, 36). Because of no heterogeneity (*I*^2^ = 0%, *P* = 0.66), a fixed-effect model was applied. The pooled result showed that the MPS cream as an add-on treatment could significantly decrease the skin inflammatory reactions of TAC-O (RR = 0.33, 95% CI: 0.17–0.66, *P* = 0.002) (Figure 7).

### Sensitivity Analysis

The leave-one-out method was conducted to evaluate the stability of the above outcomes. The pooled results did not significantly change when removing one study at a time. In addition, odds ratio (OR) was also used to calculate dichotomous data, and there was no significant difference in the pooled results of TER, recurrence rate, and skin AEs between RR measures and OR measures (Supplementary Figures 5–7). These results showed that the pooled results were relatively robust.

### Publication Bias

**Figure 8:**
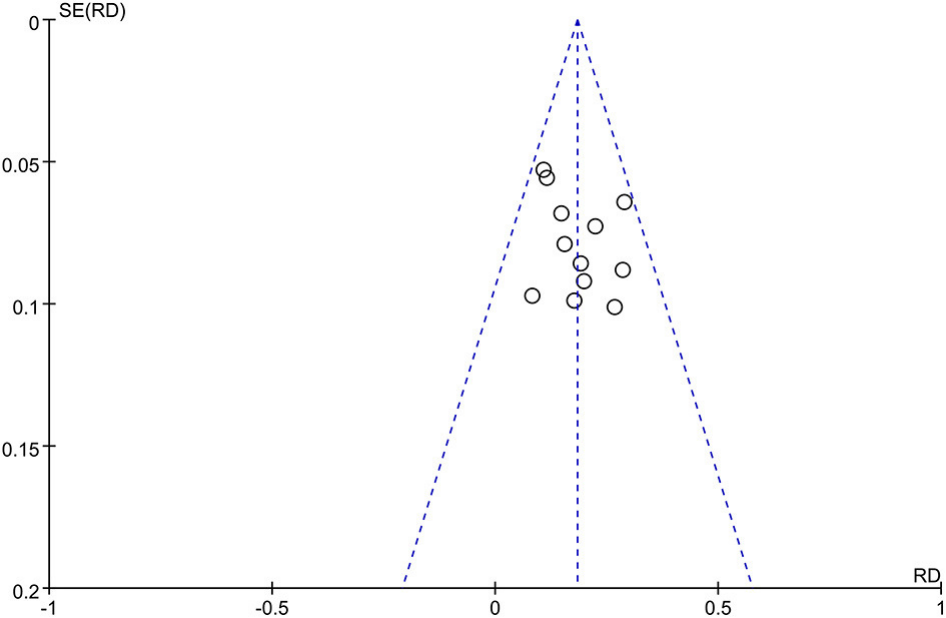
The funnel plot of publication bias for total efficacy rate between MPS cream combined with TCS and TCS monotherapy.

Publication bias on the TER between MPS cream combined with TCS and TCS alone was assessed by using the funnel plot. The nearly symmetrical funnel plot suggested no obvious publication bias (Figure 8).

## Discussion

MPS cream has been widely used for the treatment of eczema for decades, and a lot of clinical trials have confirmed its efficacy and safety. However, it is still short of an indication for eczema. Therefore, this meta-analysis collected 20 eligible studies with a relatively high number of patients and demonstrated that MPS cream as monotherapy or add-on therapy was effective with an acceptable safety profile in patients with non-exudative eczema.

The pathogenesis of eczema is complex, including epidermal barrier dysfunction, immune dysregulation, and alteration of the microbiome. Some studies on mice models showed that MPS cream could treat eczema through multiple mechanisms. First, MPS cream could restore the skin barriers by reducing TEWL and improving stratum corneum hydration. It not only elevated the expression levels of epidermal mRNA for lipid production, such as HMGCoA, fatty acid synthase (FAS), and serine palmitoyltransferase 1 (SPT1), but also increased the expression levels of some skin proteins, including filaggrin, involucrin, and loricrin (37, 38). Furthermore, when combined with TCS, MPS cream could largely prevent TCS-induced elevation in TEWL in comparison to TCS alone (39), which was also consistent with the result of this meta-analysis (21). Second, MPS cream also showed some anti-inflammatory effects. It could not only decrease total IgE and thymic stromal lymphopoietin (TSLP) in serum but also reduce the infiltration of mast cells and CD3^+^ T cells in the lesions. Moreover, the mRNA expression levels of some cytokines in lesions were also significantly decreased, including IL-4, IL-6, IL-13, and IL-22 (37). MPS cream also could suppress IL-1ß production from keratinocytes by inhibiting EKR and p38 MAPK pathways (40). In this meta-analysis, the results supported that MPS cream as an add-on treatment could significantly enhance the anti-inflammatory effect of TCS or TAC-O (25, 34). Finally, one study showed that MPS cream could upregulate the mRNA expression of mouse beta-defensin 3 (mBD3) in the epidermis, an antimicrobial peptide against Gram-negative bacteria and Candida, which demonstrated its ability to improve skin infection (38). Therefore, MPS cream could be effective in the management of eczema by improving epidermal barrier function, suppressing the inflammation of skin lesions, and enhancing antimicrobial function.

In clinical practice, emollient therapy is important and necessary to reduce lesions and delay recurrence. From the results of this meta-analysis, MPS cream significantly improved the TER and decreased recurrence rate in comparison with vaseline ointment and urea cream, suggesting that MPS cream monotherapy is effective for non-exudative eczema. On the other hand, MPS cream combined with TCS or TAC-O performed better than TCS or TAC-O alone in terms of TER and TSS. Moreover, MPS cream in combination with TCS had an advantage over TCS alone in delaying disease recurrence. However, there was no significant difference between MPS cream plus TAC-O and TAC-O alone in the recurrence rate, which may be attributed to the small number of eczema patients. These results supported the efficacy of MPS cream as an add-on treatment for non-exudative eczema.

Itch is the characteristic symptom of eczema. Although the exact pathogenesis remains unclear, some recent studies have shown that hyperinnervation of the epidermis and some itch mediators are involved, including IL-4, IL-13, IL-31, and substance P (41, 42). This meta-analysis showed that MPS cream significantly decreased pruritus score compared with vaseline ointment, indicating its effect on relieving itch. It may be attributed to that MPS cream could suppress the production of some cytokines, such as IL-4 and IL-13, and decrease the number of intraepidermal nerve fibers and the level of nerve growth factor (NGF) in the epidermis (43). Meanwhile, this meta-analysis also displayed that MPS cream twice or three times daily could significantly relieve itch, but MPS cream once daily did not achieve it. This suggested that higher antipruritic effects might be obtained if MPS cream was applied more frequently. Some studies also supported that twice-daily application of hirudoid preparations was more effective than once-daily application (44, 45). Therefore, MPS cream is recommended to be applied twice daily or more in clinical practice.

In terms of safety, this meta-analysis showed that MPS cream was well-tolerated. Only a few mild skin inflammatory reactions were reported in the MPS cream groups, such as erythema, burning, and tingling at the application site. Many clinical trials also had the same results that no systematic AEs were related to the MPS cream (46, 47). With regard to the incidence of skin inflammatory reactions, MPS cream was comparable to vaseline ointment and was superior to urea cream. On the other hand, the combination of MPS cream and TCS did not significantly increase the risk of skin inflammatory reactions compared with TCS alone, and MPS cream as an add-on treatment could reduce skin inflammatory reactions in patients treated with TAC-O. Therefore, MPS is a safe moisturizer for eczema with tolerable skin AEs. However, because patching testing was not conducted in all included studies, it is unable to know whether the skin inflammatory reactions have an allergic or irritant etiology. As two pieces of research showed, most patients allergic to MPS cream were allergic to the cream base, and the common allergens included myristyl alcohol, ceostearyl alcohol, and parabens (48, 49). Thus, people allergic to these allergens should be cautious when applying MPS cream.

There were some limitations in this meta-analysis. First, some comparisons had limited patients and studies, which led to less precise estimates. Second, the qualities of included studies were suboptimal. Most studies did not provide information on randomization and blinding, and the reliability of the results was decreased. Third, all included studies were conducted in China and only Chinese patients were enrolled. Therefore, the conclusions on MPS cream for non-exudative eczema could not be generalized directly to other countries and races. Finally, there was a lack of data on the long-term efficacy and safety of MPS cream beyond 8 weeks. Continuous application of moisturizers is useful to maintain the remission of eczema. Therefore, more long-term and well-designed studies are required to explore the safety and efficacy of MPS cream in the maintenance treatment of eczema.

## Conclusion

In conclusion, this meta-analysis demonstrated that MPS cream as monotherapy or add-on therapy could provide a good effect for the treatment of non-exudative eczema with mild and tolerable skin reactions. However, the findings should be interpreted carefully because of the suboptimal quality and limited sample size. More high-quality and large-sample RCTs are needed in the future for validation and update.

## Data Availability Statement

The original contributions presented in the study are included in the article/Supplementary Material, further inquiries can be directed to the corresponding author/s.

## Author Contributions

ML and LL were responsible for the study concept and design. ML, YL, and LX contributed to study collection, data extraction, and assessment of bias risk. ML and YL performed the data analysis and interpretation. ML drafted the manuscript. All authors contributed to the manuscript revision, read, and approved the submitted version.

## Conflict of Interest

The authors declare that the research was conducted in the absence of any commercial or financial relationships that could be construed as a potential conflict of interest.

## Publisher's Note

All claims expressed in this article are solely those of the authors and do not necessarily represent those of their affiliated organizations, or those of the publisher, the editors and the reviewers. Any product that may be evaluated in this article, or claim that may be made by its manufacturer, is not guaranteed or endorsed by the publisher.

## Abbreviations

MPS: mucopolysaccharide polysulfate
TCS: topical corticosteroids
TCI: topical calcineurin inhibitor
TAC-O: tacrolimus ointment
TER: total efficacy rate
TSS: total symptoms score
AE: adverse event
TEWL: trans-epidermal water loss
MD: mean difference
SMD: standard mean difference.
