# Supplementary material for: Efficacy and Safety of Mucopolysaccharide Polysulfate Cream for Non-Exudative Eczema: A Systematic Review and Meta-Analysis
Source: Front Med (Lausanne). 2021 Dec 24;8:788324. doi: 10.3389/fmed.2021.788324 (PMC8738087; doi:10.3389/fmed.2021.788324)
Supplement: Supplementary file 1 [file Data_Sheet_1.PDF]

Study or Subgroup	Experimental Mean	Experimental SD	Experimental Total	Control Mean	Control SD	Control Total	Weight	Mean Difference IV, Fixed, 95% CI
Yang 2016	0.34	0.12	58	2.12	1.47	59	100.0%	-1.78 [-2.16, -1.40]
**Total (95% CI)**			**58**			**59**	**100.0%**	**-1.78 [-2.16, -1.40]**

Heterogeneity: Not applicable

Test for overall effect: Z = 9.27 (P < 0.00001)

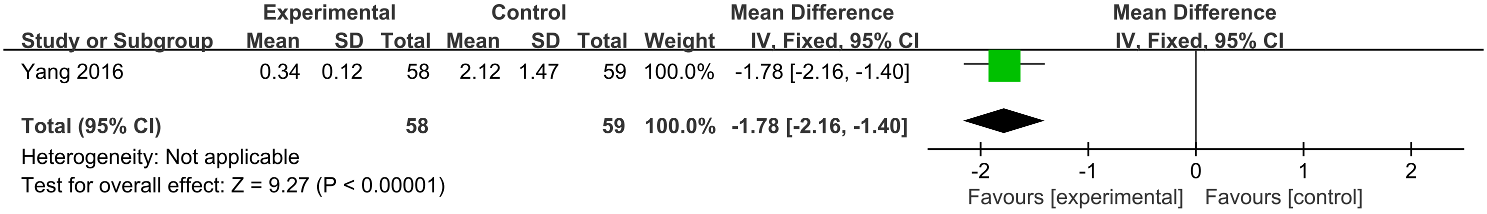

Supplementary Figure 1. The forest plot for pruritus score between MPS cream and vaseline ointment.

Study or Subgroup	Experimental Mean	Experimental SD	Experimental Total	Control Mean	Control SD	Control Total	Weight	Mean Difference IV, Fixed, 95% CI
**4.6.1 Transepidermal water loss**								
Hu et al 2019	15.25	3.18	41	19.21	4.79	41	100.0%	-3.96 [-5.72, -2.20]
**Subtotal (95% CI)**			**41**			**41**	**100.0%**	**-3.96 [-5.72, -2.20]**
Heterogeneity: Not applicable								
Test for overall effect: Z = 4.41 (P < 0.0001)								
**4.6.2 Stratum corneum hydration**								
Hu et al 2019	28.35	5.63	41	24.12	4.47	41	100.0%	4.23 [2.03, 6.43]
**Subtotal (95% CI)**			**41**			**41**	**100.0%**	**4.23 [2.03, 6.43]**
Heterogeneity: Not applicable								
Test for overall effect: Z = 3.77 (P = 0.0002)								

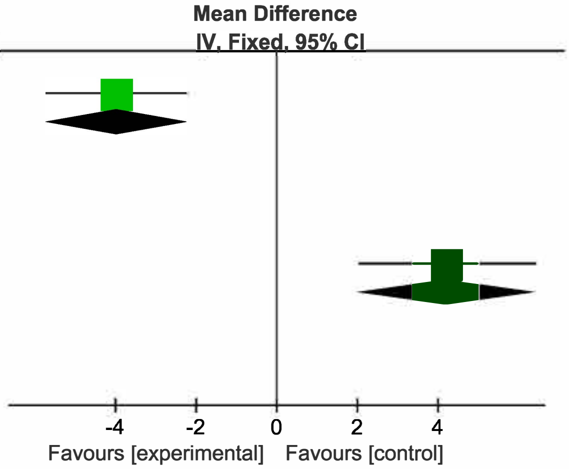

Supplementary Figure 2. The forest plot for the levels of TEWL and stratum corneum hydration between MPS cream combined with triamcinolone acetonide and econazole cream and triamcinolone acetonide and econazole cream monotherapy.

	Experimental			Control				Mean Difference
Study or Subgroup	Mean	SD	Total	Mean	SD	Total	Weight	IV, Fixed, 95% CI
**4.11.1 IL-4**								
Wang et al 2018	35.43	4.08	48	50.19	4.87	47	100.0%	-14.76 [-16.57, -12.95]
**Subtotal (95% CI)**			**48**			**47**	**100.0%**	**-14.76 [-16.57, -12.95]**
Heterogeneity: Not applicable								
Test for overall effect: Z = 16.00 (P < 0.00001)								
**4.11.2 IL-10**								
Wang et al 2018	7.35	1.49	48	10.17	1.83	47	100.0%	-2.82 [-3.49, -2.15]
**Subtotal (95% CI)**			**48**			**47**	**100.0%**	**-2.82 [-3.49, -2.15]**
Heterogeneity: Not applicable								
Test for overall effect: Z = 8.23 (P < 0.00001)								
**4.11.3 IFN-y**								
Wang et al 2018	61.34	6.18	48	52.9	5.47	47	100.0%	8.44 [6.09, 10.79]
**Subtotal (95% CI)**			**48**			**47**	**100.0%**	**8.44 [6.09, 10.79]**
Heterogeneity: Not applicable								
Test for overall effect: Z = 7.05 (P < 0.00001)								

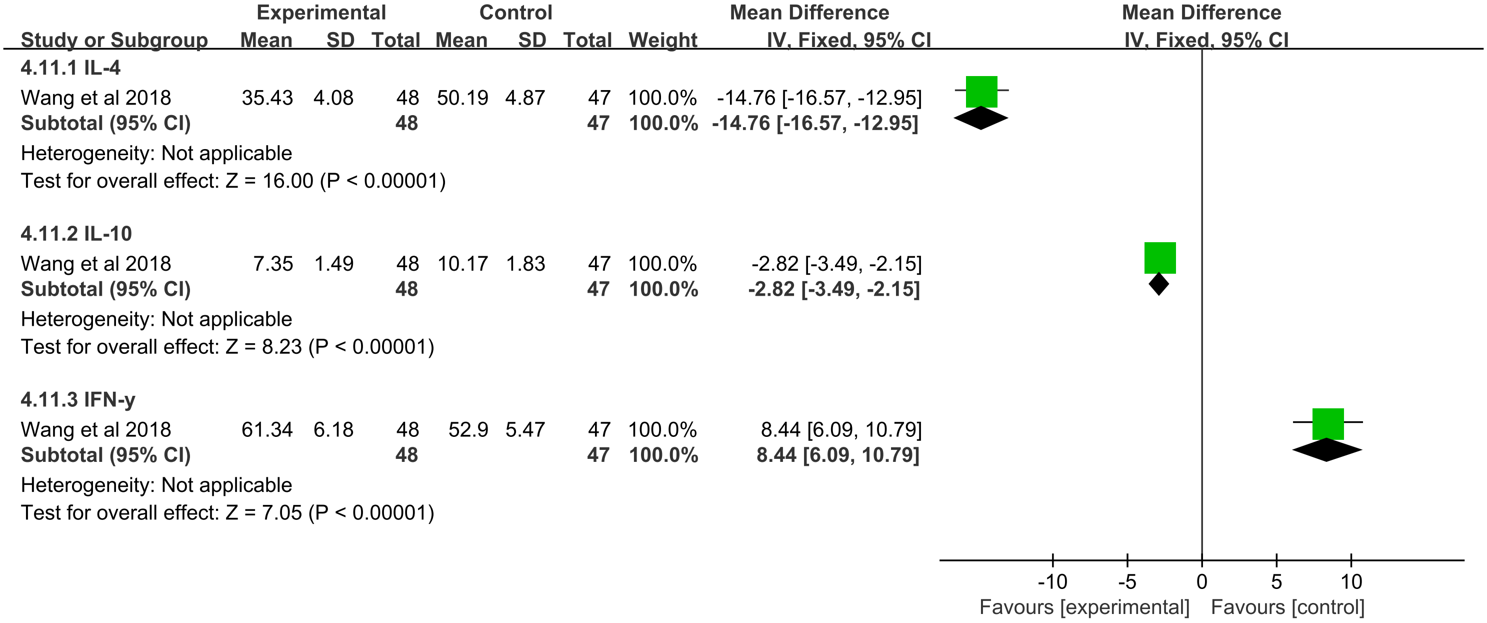

Supplementary Figure 3. The forest plot for the levels of cytokines in serum between MPS cream combined with hydrocortisone cream and hydrocortisone cream monotherapy.

Study or Subgroup	Experimental Mean	Experimental SD	Experimental Total	Control Mean	Control SD	Control Total	Weight	Mean Difference IV, Fixed, 95% CI
**4.12.1 IL-4**								
Wang 2019	32.32	7.23	43	37.15	7.15	43	100.0%	-4.83 [-7.87, -1.79]
**Subtotal (95% CI)**			**43**			**43**	**100.0%**	**-4.83 [-7.87, -1.79]**
Heterogeneity: Not applicable								
Test for overall effect: Z = 3.11 (P = 0.002)								
**4.12.2 IL-2**								
Wang 2019	45.57	10.56	43	35.63	6.75	43	100.0%	9.94 [6.19, 13.69]
**Subtotal (95% CI)**			**43**			**43**	**100.0%**	**9.94 [6.19, 13.69]**
Heterogeneity: Not applicable								
Test for overall effect: Z = 5.20 (P < 0.00001)								
**4.12.3 IFN-y**								
Wang 2019	48.58	10.57	43	42.37	8.16	43	100.0%	6.21 [2.22, 10.20]
**Subtotal (95% CI)**			**43**			**43**	**100.0%**	**6.21 [2.22, 10.20]**
Heterogeneity: Not applicable								
Test for overall effect: Z = 3.05 (P = 0.002)								

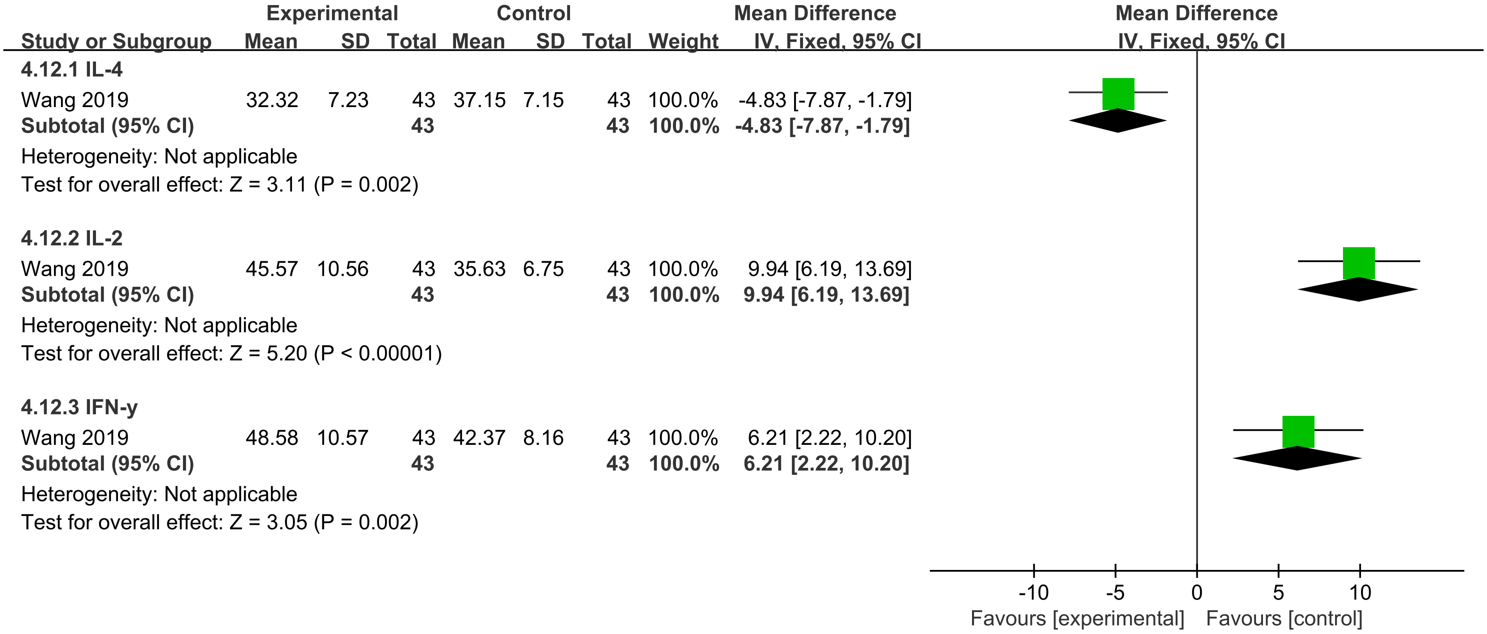

Supplementary Figure 4. The forest plot for the levels of cytokines in serum between MPS cream combined with TAC-O and TAC-O monotherapy.

Study or Subgroup	Experimental Events	Experimental Total	Control Events	Control Total	Weight	Odds Ratio M-H, Fixed, 95% CI
**1.1.1 MPS cream versus Moisturizers**						
Tan et al 2019	220	250	179	250	96.1%	2.91 [1.82, 4.65]
Yang 2016	57	58	51	59	3.9%	8.94 [1.08, 73.96]
**Subtotal (95% CI)**		**308**		**309**	**100.0%**	**3.14 [1.99, 4.96]**
Total events	277		230			
Heterogeneity: Chi² = 1.05, df = 1 (P = 0.31); I² = 4%						
Test for overall effect: Z = 4.93 (P < 0.00001)						
**1.1.2 MPS cream combined with TCS versus TCS**						
Dang et al 2019	77	90	51	90	11.8%	4.53 [2.20, 9.31]
Feng 2014	54	60	45	60	7.2%	3.00 [1.08, 8.37]
Guo 2016	39	40	30	40	1.2%	13.00 [1.58, 107.23]
Hu et al 2019	32	41	21	41	7.4%	3.39 [1.30, 8.85]
Liu 2018	33	49	29	49	15.2%	1.42 [0.62, 3.25]
Liu and Liu 2014	50	52	23	31	1.8%	8.70 [1.71, 44.22]
Shi 2019	42	46	31	41	4.6%	3.39 [0.97, 11.81]
Wang and Guo 2018	88	100	77	100	14.8%	2.19 [1.02, 4.69]
Wang et al 2018	33	48	24	47	12.2%	2.11 [0.91, 4.87]
Xiao et al 2012	33	36	23	32	3.3%	4.30 [1.05, 17.65]
Xu et al 2016	52	60	41	60	8.8%	3.01 [1.20, 7.57]
Zhang et al 2014	33	35	24	32	2.3%	5.50 [1.07, 28.25]
Zhang et al 2020	29	43	18	43	9.4%	2.88 [1.19, 6.93]
**Subtotal (95% CI)**		**700**		**666**	**100.0%**	**3.07 [2.34, 4.03]**
Total events	595		437			
Heterogeneity: Chi² = 10.16, df = 12 (P = 0.60); I² = 0%						
Test for overall effect: Z = 8.11 (P < 0.00001)						
**1.1.3 MPS cream combined TAC-O versus TAC-O**						
Di and Xu 2014	25	30	21	28	16.2%	1.67 [0.46, 6.03]
Dong 2020	22	48	16	48	38.8%	1.69 [0.74, 3.87]
Wang 2019	37	43	29	43	18.1%	2.98 [1.02, 8.70]
Xiang 2017	38	50	25	50	26.9%	3.17 [1.35, 7.44]
**Subtotal (95% CI)**		**171**		**169**	**100.0%**	**2.32 [1.44, 3.74]**
Total events	122		91			
Heterogeneity: Chi² = 1.53, df = 3 (P = 0.67); I² = 0%						
Test for overall effect: Z = 3.44 (P = 0.0006)						

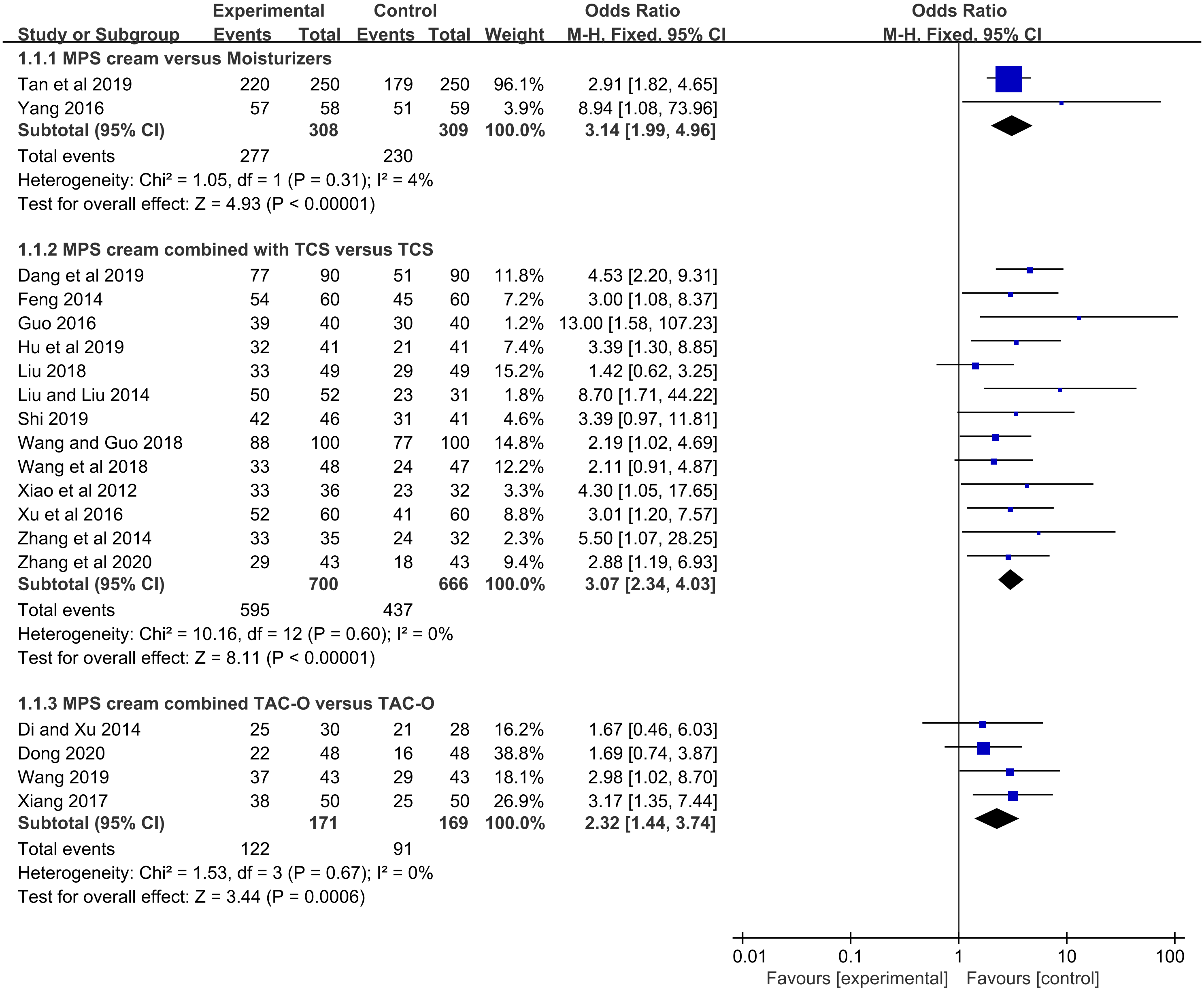

Supplementary Figure 5. The forest plot for total efficacy rate calculated by odds ratio between MPS cream therapy and non-MPS cream therapy.

Study or Subgroup	Experimental Events	Experimental Total	Control Events	Control Total	Weight	Odds Ratio M-H, Fixed, 95% CI
**1.2.1 MPS cream versus Urea cream**						
Tan et al 2019	18	138	30	101	100.0%	0.35 [0.18, 0.68]
**Subtotal (95% CI)**		**138**		**101**	**100.0%**	**0.35 [0.18, 0.68]**
Total events	18		30			
Heterogeneity: Not applicable						
Test for overall effect: Z = 3.10 (P = 0.002)						
**1.2.2 MPS cream combined with TCS versus TCS**						
Feng 2014	6	49	15	38	100.0%	0.21 [0.07, 0.63]
**Subtotal (95% CI)**		**49**		**38**	**100.0%**	**0.21 [0.07, 0.63]**
Total events	6		15			
Heterogeneity: Not applicable						
Test for overall effect: Z = 2.81 (P = 0.005)						
**1.2.3 MPS cream combined with TAC-O versus TAC-O**						
Wang 2019	5	28	7	17	100.0%	0.31 [0.08, 1.22]
**Subtotal (95% CI)**		**28**		**17**	**100.0%**	**0.31 [0.08, 1.22]**
Total events	5		7			
Heterogeneity: Not applicable						
Test for overall effect: Z = 1.68 (P = 0.09)						

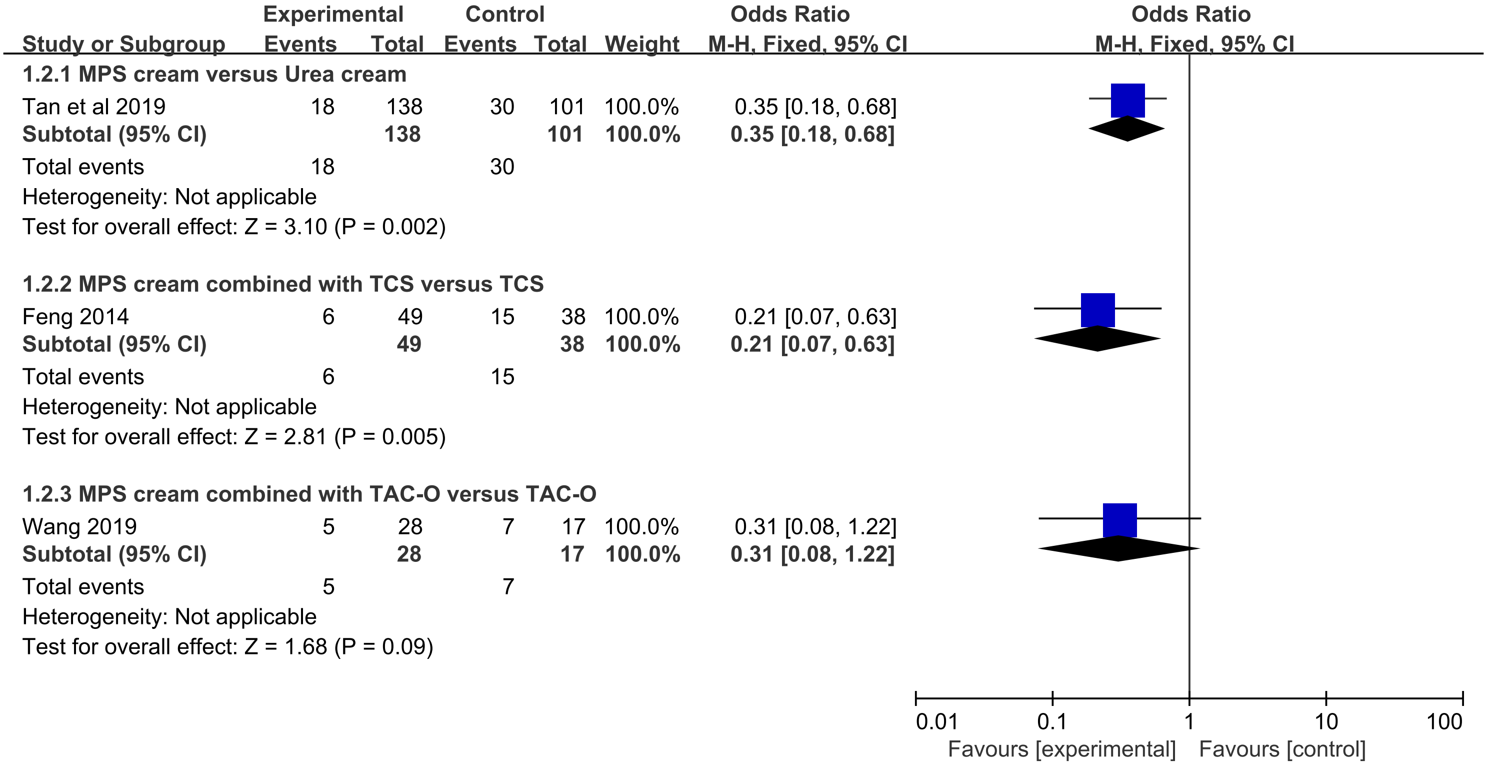

Supplementary Figure 6. The forest plot for recurrence rate calculated by odds ratio between MPS cream therapy and non-MPS cream therapy.

Study or Subgroup	Experimental Events	Experimental Total	Control Events	Control Total	Weight	Odds Ratio M-H, Fixed, 95% CI
**1.4.1 MPS cream versus Urea cream**						
Tan et al 2019	2	250	10	250	100.0%	0.19 [0.04, 0.89]
**Subtotal (95% CI)**		**250**		**250**	**100.0%**	**0.19 [0.04, 0.89]**
Total events	2		10			
Heterogeneity: Not applicable						
Test for overall effect: Z = 2.11 (P = 0.04)						
**1.4.2 MPS cream versus Vaseline ointment**						
Yang 2016	1	58	2	59	100.0%	0.50 [0.04, 5.67]
**Subtotal (95% CI)**		**58**		**59**	**100.0%**	**0.50 [0.04, 5.67]**
Total events	1		2			
Heterogeneity: Not applicable						
Test for overall effect: Z = 0.56 (P = 0.58)						
**1.4.3 MPS cream combined with TCS versus TCS**						
Feng 2014	0	60	0	60		Not estimable
Guo 2016	0	40	3	40	54.1%	0.13 [0.01, 2.65]
Liu 2018	1	49	2	49	30.7%	0.49 [0.04, 5.58]
Shi 2019	1	46	0	41	8.0%	2.74 [0.11, 69.04]
Wang and Guo 2018	0	100	0	100		Not estimable
Xiao et al 2012	0	36	0	32		Not estimable
Zhang et al 2014	0	35	0	32		Not estimable
Zhang et al 2020	3	43	0	43	7.2%	7.52 [0.38, 150.10]
**Subtotal (95% CI)**		**409**		**397**	**100.0%**	**0.98 [0.33, 2.96]**
Total events	5		5			
Heterogeneity: Chi² = 4.20, df = 3 (P = 0.24); I² = 29%						
Test for overall effect: Z = 0.03 (P = 0.97)						
**1.4.4 MPS cream combined with TAC-O versus TAC-O**						
Di and Xu 2014	4	30	10	30	36.2%	0.31 [0.08, 1.13]
Xiang 2017	5	50	17	50	63.8%	0.22 [0.07, 0.64]
**Subtotal (95% CI)**		**80**		**80**	**100.0%**	**0.25 [0.11, 0.57]**
Total events	9		27			
Heterogeneity: Chi² = 0.17, df = 1 (P = 0.68); I² = 0%						
Test for overall effect: Z = 3.27 (P = 0.001)						

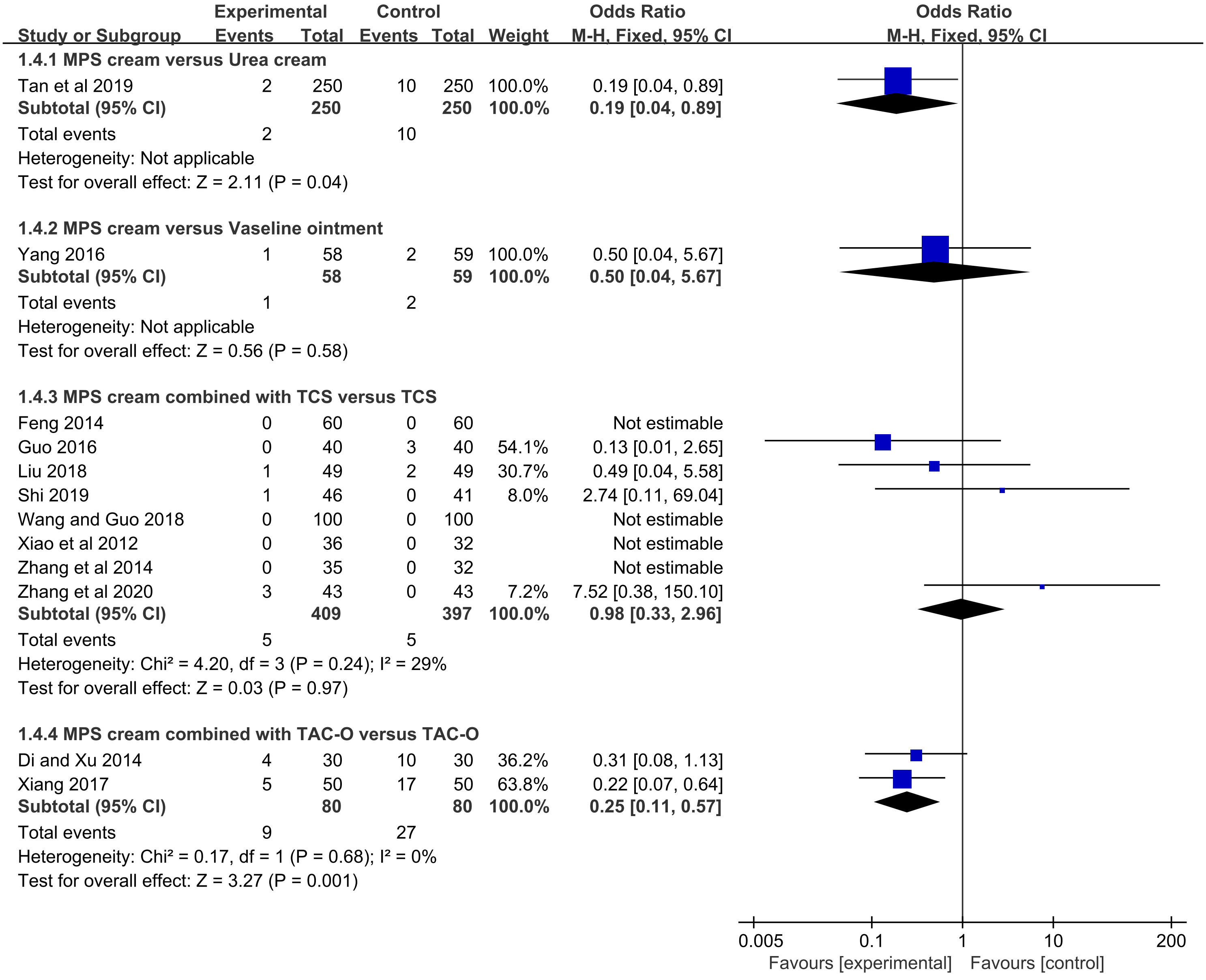

Supplementary Figure 7. The forest plot for the incidence of skin inflammatory reactions calculated by odds ratio between MPS cream therapy and non-MPS cream therapy.
